# Cochlear Implant After Sudden Onset Sensorineural Hearing Loss: A Case Report

**DOI:** 10.7759/cureus.35559

**Published:** 2023-02-27

**Authors:** Deven Curtis, Avi Shaw, Noorullah Z Malik, Anthony Baumann, Anita Jeyakumar

**Affiliations:** 1 Otolaryngology, Northeast Ohio Medical University, Rootstown, USA; 2 Otolaryngology - Head and Neck Surgery, Mercy Health, Youngstown, USA; 3 Biomedical Sciences, The University of Akron, Akron, USA; 4 Rehabilitation Services, University Hospitals Cleveland Medical Center, Cleveland, USA

**Keywords:** chatgpt, chat gpt, sudden onset hearing loss, sudden hearing loss, cochlear implant, pediatric hearing loss, otolaryngology, sudden sensorineural hearing loss

## Abstract

We present an uncommon case of a pediatric patient with sudden-onset sensorineural hearing loss (SSNHL), a medical condition in which a person experiences a rapid loss of 30 or more decibels within a matter of hours or days. The patient is a nine-year-old female who, two years prior, suddenly lost hearing in her left ear after a 24-hour episode of nausea, vomiting, and left ear pain. She presented to our clinic two years after the episode, long after the window for evidence-based treatment for acute SSNHL, such as corticosteroid therapy or antivirals, had passed. However, she remembered the moment of her hearing loss vividly, an uncommon occurrence in pediatric patients. CT, MRI, family history, and physical exam were unremarkable. The patient had a brief hearing aid trial where she described being able to hear the sound but did not have any clarity in understanding the sound. The patient was ultimately treated with a unilateral cochlear implant and showed excellent subjective and audiogram responses. Continued research on the management of SSNHL in pediatric patients who present outside of the acute therapeutic window is needed.

## Introduction

Sudden onset sensorineural hearing loss (SSNHL) is a measurable unilateral or bilateral hearing impairment lasting less than 72 hours [1]. The incidence of SSNHL is considered to be rare, with an estimated occurrence rate of around 5-30 cases per 100,000 individuals per year in the United States [1-2]. It is more common in men than women and tends to occur in individuals between the ages of 30 and 60, with incidence increasing with age [2]. Rarely, it can affect children, but such reports are relatively sparse, usually have no known etiology, and hearing is seldom restored [3]. The paucity of information in the literature regarding the management of these cases leaves providers and patients with many unanswered questions regarding best practices and proven treatment options for pediatric SSNHL, particularly after the acute therapeutic window has passed. The purpose of this study is to exemplify the diagnosis and management of chronic SSNHL in a pediatric patient via cochlear implantation.

## Case presentation

**Figure 1:**
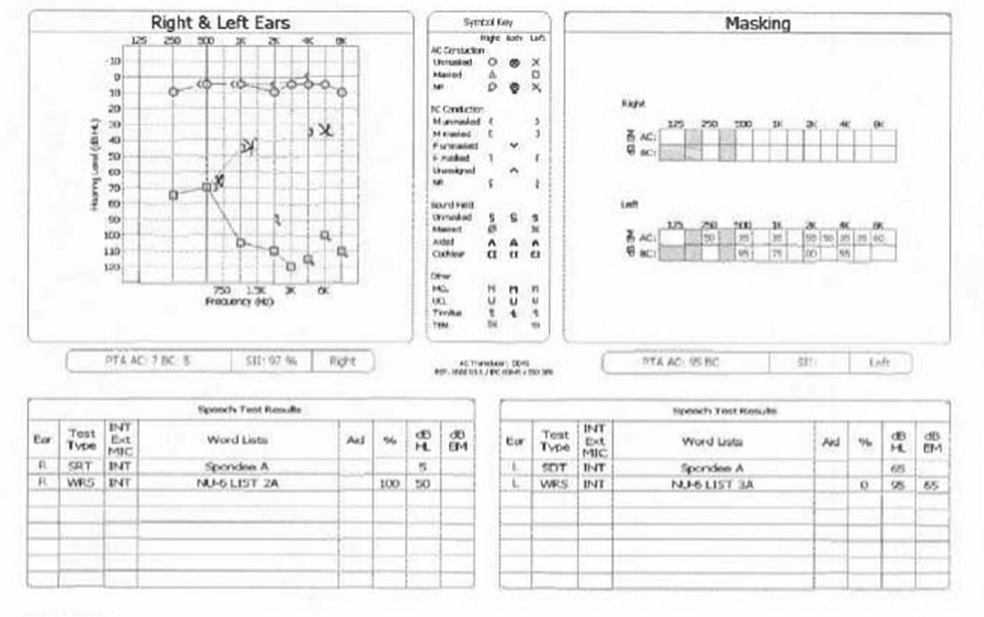
At the first office visit, a pure tone audiogram showed normal right ear hearing with profound hearing loss of the left ear.

A nine-year-old female presented to the clinic accompanied by her father with complaints of chronic left-sided hearing loss. The patient reported that two years prior, she had been swimming and experienced a sudden episode of otalgia with associated nausea and vomiting, which lasted for about 24 hours and resolved spontaneously but was followed by a subjective sense of left hearing loss. Her father reported that the patient had been less “attentive” over the past two years, which finally prompted them to be evaluated. Upon evaluation via a hearing screen and audiogram, she was found to have a profound hearing loss in the left ear with normal hearing on the right (Figure 1).

**Figure 2:**
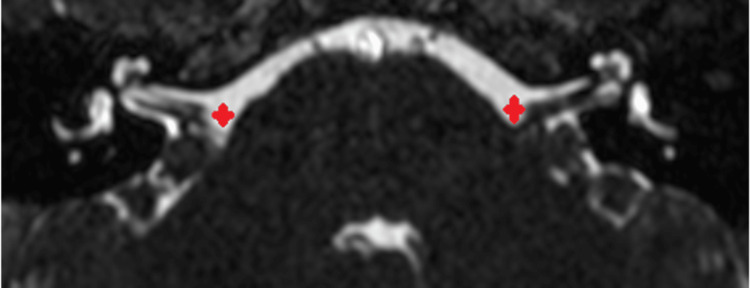
MRI with internal auditory canal protocol demonstrating normal anatomy of cranial nerves VIII and VII (marked in red).

**Figure 3:**
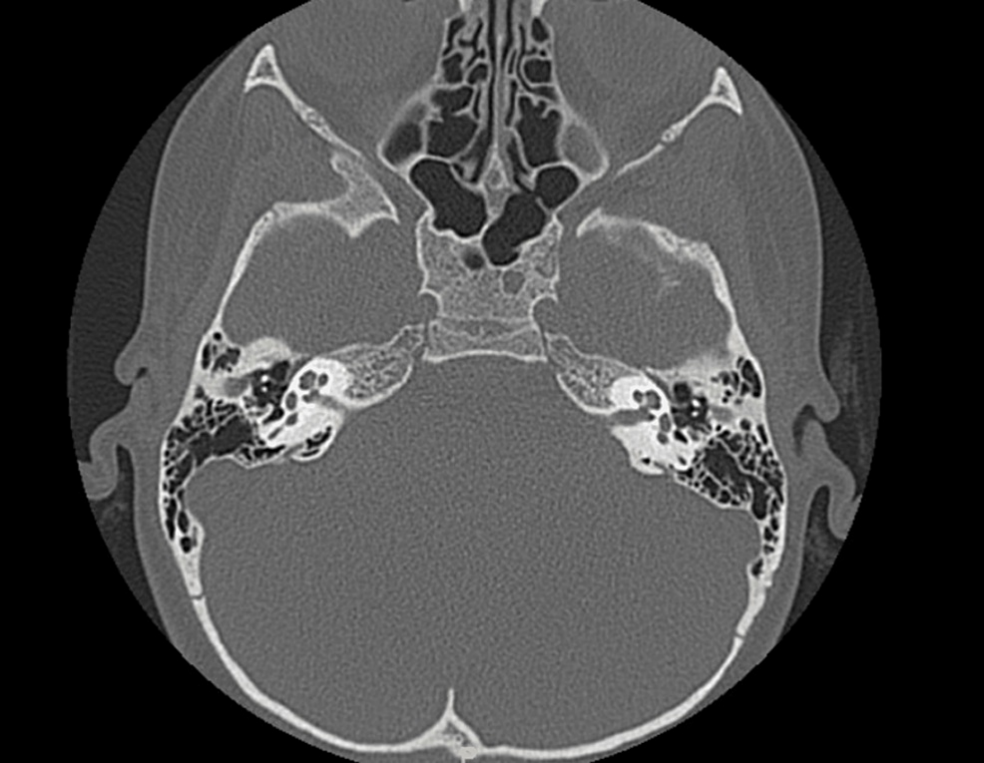
High resolution axial CT IAC without contrast shows bilateral middle ears without abnormalities. IAC: Internal auditory canal.

The patient is an only child with no known family history of hearing loss. She was born healthy at full term, and she passed her newborn hearing screen. A physical exam revealed a well-developed nine-year-old female with unremarkable findings, including an intact tympanic membrane and surrounding structures. MRI (Figure 2) and CT (Figure 3) were obtained to rule out potential causes such as hypoplasia, cranial etiology, or neoplasm, and both studies showed normal anatomy with no abnormalities.

**Figure 4:**
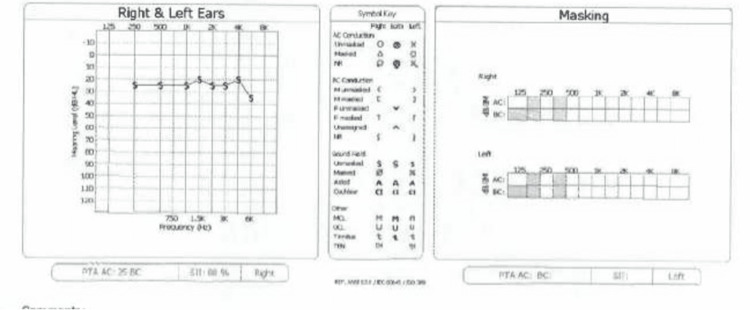
Pure tone audiogram of left ear status post cochlear implant.

Physical exams, imaging findings, and treatment options were discussed with the patient and medical decision-makers. The possible treatment options offered were continued observation with no intervention, using a contralateral routing of signals (CROS) hearing aid, a bone conduction hearing implant (Baha), and a cochlear implant. Ultimately, the patient and family elected a cochlear implant after the shared decision-making process and an unsatisfactory hearing aid trial. High-resolution computed tomography (HRCT) was referenced for surgical planning. A commercially available, FDA-approved implant was selected, and surgical placement was successful and without incident. Recovery followed the typical course, and since the time of the implant, the patient follow-up visits have shown excellent response to the implant, and the patient is doing well and is satisfied with the outcomes. Audiogram shows significant improvement from pre-operative measurements, with hearing measuring within normal levels (Figure 4).

## Discussion

Symptoms of SSNHL include a sudden loss of hearing, tinnitus, and vertigo. The cause of SSNHL is most often not well understood and is only identified in 10% of all cases [3]. Among known causes, there are multiple implicated factors, including autoimmune disorders and viral infections, for example, in recent years, COVID-19 infection [4]. Other factors that may increase the risk of developing SSNHL include exposure to loud noises, certain medical conditions (such as Meniere's disease, autoimmune disorders, and diabetes), and certain medications (such as aminoglycoside antibiotics and cisplatin). Other areas currently under study recommendation within the scope of pediatric SSNHL are the association of SSNHL incidence and low fasting glucose levels and complement proteins [5]. Among viral etiologies, cytomegalovirus frequently appears in the literature and has been found, in at least one large study, to have an association with 34% of pediatric SSNHL cases [6]. The prevalence of SSNHL is relatively low in the general population and even lower in the pediatric population. The exact prevalence rate is difficult to determine as it depends on factors such as population size and age, the definition of sudden hearing loss, and data collection methods. However, some estimates suggest that the prevalence of SSNHL may be as low as 1-2 cases per 100,000 individuals per year [7]. It is also worth noting that the prevalence of SSNHL may be underreported as some people may delay medical evaluation and treatment.

Most current literature suggests the management of SSNHL with steroids such as prednisone, which may be prescribed to reduce inflammation and swelling in the inner ear to improve or stabilize hearing. While these can be systemically administered, some data suggests that intratympanic steroid therapy can be more effective than oral steroids, especially when administered at higher doses [8]. Transtympanic steroid injections have been shown to be an effective therapy option in at least partial hearing recovery of about 50% of patients when given in the acute window. They remain far and away the most common form of treatment for SSNHL patients [6, 9]. There have been heterogeneous data about other acute treatment options, including antiviral and immunoglobulin therapy, all with variable results and outcomes. Similarly, in such cases where auto-immune disorder is suspected, plasmapheresis or immunoglobulin therapy may be recommended. However, this is seldom performed, and little exists in the body of literature substantiating this treatment method. While these are considerations during the acute window, these were not considered in our case. While corticosteroids are a mainstay of treatment and have been shown to improve patient outcomes, they are indicated only for acute management, which is generally considered to be on the order of days to weeks, not months to years. This creates a narrow treatment window, especially because pediatric patients do not often present within the timeframe and are thus relegated to non-steroidal treatment options, none of which have highly favorable outcomes. Such was the case with our patient, who suffered from SSNHL but was not evaluated until two years post-onset, presumably long after the cessation of the inciting insult. Providers faced with managing profound single-sided hearing loss in pediatric patients, specifically those who lost the hearing acutely, have no abnormal imaging, and are present for chronic management, have little evidence-based guidance on restoring hearing to the affected ear. CROS hearing aids and Baha implants may be compensatory therapies that provide some recourse and may reduce the residual disability. However, they accomplish little in the way of addressing the underlying issue and restoring sound transmission in the affected ear. Cochlear implantation, however, offers the potential recovery of the function of the affected ear, as was the case with our patient, and may represent an underutilized treatment path that could offer similar patients another viable treatment option. More research is needed to manage chronic SSNHL to improve patient outcomes and prevent complications.

## Conclusions

SSNHL is a complex, multifactorial, and poorly understood condition facing patients and providers. The literature offers little recourse for providers treating late management of SSNHL in pediatric patients where the window for the mainstay of treatment, namely transtympanic corticosteroid therapy, has passed. To this end, we present a pediatric patient who suffered profound unilateral SSNHL two years prior to presentation and demonstrated an excellent response to cochlear implantation.

## References

[1] Chandrasekhar SS, Tsai Do BS, Schwartz SR (2019). Clinical practice guideline: sudden hearing loss (update). Otolaryngol Head Neck Surg.

[2] Alexander TH, Harris JP (2013). Incidence of sudden sensorineural hearing loss. Otol Neurotol.

[3] Penido NO, Cruz OL, Zanoni A, Inoue DP (2009). Classification and hearing evolution of patients with sudden sensorineural hearing loss. Braz J Med Biol Res.

[4] Yaseen NK, Al-Ani RM, Ali Rashid R (2021). COVID-19-related sudden sensorineural hearing loss. Qatar Med J.

[5] Lu Y, Zhou L, Imrit TS, Liu A (2019). Sudden sensorineural hearing loss in children: clinical characteristics, etiology, treatment outcomes, and prognostic factors. Otol Neurotol.

[6] Wood JW, Shaffer AD, Kitsko D, Chi DH (2021). Sudden sensorineural hearing loss in children-management and outcomes: a meta-analysis. Laryngoscope.

[7] Naz E, Saqulain G, Mumtaz N, Babur MN (2021). A Hospital based study on sudden sensorineural Hearing Loss: It's audiological characteristics and prevalence. Pak J Med Sci.

[8] Li J, Ding L (2020). Effectiveness of steroid treatment for sudden sensorineural hearing loss: a meta-analysis of randomized controlled trials. Ann Pharmacother.

[9] Marx M, Younes E, Chandrasekhar SS, Ito J, Plontke S, O'Leary S, Sterkers O (2018). International consensus (ICON) on treatment of sudden sensorineural hearing loss. Eur Ann Otorhinolaryngol Head Neck Dis.

